# The Model of Interstitial Cystitis for Evaluating New Molecular Strategies of Interstitial Regeneration in Humans

**DOI:** 10.3390/ijms25042326

**Published:** 2024-02-15

**Authors:** Elisabetta Mormone, Antonio Cisternino, Lorenzo Capone, Eugenio Caradonna, Andrea Sbarbati

**Affiliations:** 1 Intitute for Stem-Cell Biology, Regenerative Medicine and Innovative Therapies (ISBReMIT), Fondazione IRCCS Casa Sollievo della Sofferenza, Viale dei Cappuccini 1, 71013 San Giovanni Rotondo, Italy; 2 Santa Maria di Bari Hospital, Via Antonio de Ferraris 22, 70124 Bari, Italy; antonio.cisternino@libero.it; 3 Department of Urology, Fondazione IRCCS Casa Sollievo della Sofferenza, Viale dei Cappuccini 1, 71013 San Giovanni Rotondo, Italy; lorenzocapone@msn.com; 4 Gemelli Molise S.p.a., Largo Gemelli 1, 86100 Campobasso, Italy; eugenio.caradonna@gmail.com; 5 Department of Neuroscience, Biomedicine and Movement Sciences, Human Anatomy and Histology Section, University of Verona, 37129 Verona, Italy; andrea.sbarbati@univr.it

**Keywords:** hyaluronic acid, interstitial cystitis, mesenchymal stem cells, nitric oxide, PRP, regenerative medicine, SVF

## Abstract

Given the recent evidence in the clinical application of regenerative medicine, mostly on integumentary systems, we focused our interests on recent bladder regeneration approaches based on mesenchymal stem cells (MSCs), platelet-rich plasma (PRP), and hyaluronic acid (HA) in the treatment of interstitial cystitis/bladder pain syndrome (IC/BPS) in humans. IC/BPS is a heterogeneous chronic disease with not-well-understood etiology, characterized by suprapubic pain related to bladder filling and urothelium dysfunction, in which the impairment of immunological processes seems to play an important role. The histopathological features of IC include ulceration of the mucosa, edema, denuded urothelium, and increased detection of mast cells and other inflammatory cells. A deeper understanding of the molecular mechanism underlying this disease is essential for the selection of the right therapeutic approach. In fact, although various therapeutic strategies exist, no efficient therapy for IC/BPS has been discovered yet. This review gives an overview of the clinical and pathological features of IC/BPS, with a particular focus on the molecular pathways involved and a special interest in the ongoing few investigational therapies in IC/BPS, which use new regenerative medicine approaches, and their synergetic combination. Good knowledge of the molecular aspects related to stem cell-, PRP-, and biomaterial-based treatments, as well as the understanding of the molecular mechanism of this pathology, will allow for the selection of the right and best use of regenerative approaches of structures involving connective tissue and epithelia, as well as in other diseases.

## 1. Introduction

In recent years, regenerative medicine has reached significant achievements, specifically in terms of the employment of stem cells and growth factors. The wide clinical application of regenerative medicine is due to the development of both simple and effective technologies to gain autologous preparations, capable of modifying structures affected by lesions or simply aged tissue. Among stem cells, mesenchymal cells have proved to be very versatile, in particular for their ease of collection from various organs. Among growth factors, a relevant role is represented by platelet growth factors, whose easy sampling has played a role favoring their huge clinical use. These products act in particular on connective tissue, but their effects extend to associate epithelial tissues as well, with mechanisms still to be completely understood. These data are confirmed by a large amount of studies in the literature referring mainly to the integumentary system (i.e., both skin and subcutaneous tissue), while studies on visceral structures, and in particular hollow organs, appear to be more limited, although these, for their anatomical and histological characteristics, as well as in the clinic, would be very similar to integumentary tissues. In particular, regenerative medicine studies on the urinary tract are poor, but the data available so far suggest that regenerative technologies could be of great utility, also considering the great social impact of the pathologies of this district. Furthermore, the aforementioned applications pose new challenges to tissue analysis techniques, such as histology, histochemistry, molecular, and tissue biology, as the evaluation of treatments requires protocols adapted to new technologies. In the present work, we analyzed data related to interstitial cystitis, which can be considered an excellent pathology model for the connective tissue of a hollow viscera and for which protocols based on compounds, similar to those used in the integumentary system, such as hyaluronic acid, have already been in place for long time. Particularly, we focused our interest on the few ongoing clinical trials based on the employment of MSCs and PRP that have already been widely applied in orthopedics, dermatology, and ophthalmology as healing therapy. Therefore, interstitial cystitis may be considered to be a good example for developing innovative strategies aimed at the regeneration of connective tissues and associated epithelia. However, the complexity of regenerative approaches requires a detailed molecular and cellular analysis of aspects related to stem cell, PRP, and biomaterial treatments, an analysis that is frequently lacking from the scientific literature.

## 2. Interstitial Cystitis/Bladder Pain Syndrome

### 2.1. IC/BPS Definition and Evaluation

The American Urological Association (AUA) describes interstitial cystitis/bladder pain syndrome (IC/BPS) as a heterogeneous, chronic (>6 weeks duration), and poorly understood pelvic condition, characterized by suprapubic pain, discomfort, or pressure related to bladder filling [1]. The condition is characterized by chronic inflammation and other symptoms, such as increased daytime or nighttime frequency, in the absence of proven urinary tract infection or other obvious bladder pathology [2,3,4]. It is difficult to define both the pathophysiological mechanisms and the therapeutic strategies used to obtain symptom alleviation. Symptom severity is exclusive for each individual, and some patients may experience symptom-free intervals [5]. The analysis is difficult and is deemed a diagnosis of exclusion. Based totally on cystoscopic findings, patients with IC are categorized as either Hunner-type/classic IC and non-Hunner-type IC. Hunner-type IC offers a “circumscript, reddened mucosal lesion with small vessels radiating towards a central scar, with a fibrin deposit or coagulum” attached to this area [6]. Non-Hunner-type IC does not present typical lesions, apart from posthydro distension mucosal bleeding. Under cystoscopy, in fact, the presence of submucosal petechial bleeding, so-called glomerulations, after decompression of the previously distended bladder, has been regarded as one of the endoscopic hallmarks of the disease, together with Hunner’s lesions [7]. Bladder biopsies are not routinely necessary or recommended according to the AUA guidelines unless malignancy is suspected [8]. In both Hunner and non-Hunner IC/BPS, cystoscopic and biopsy findings indicate defects in the urothelial glycosaminoglycan (GAGs) layer, which may expose, if damaged, submucosal nerve filaments to irritative urine components [2].

### 2.2. IC/BPS Etiology and Pathophysiology

The etiology of IC/BPS is not well understood yet, as multiple effects may manifest as a final common bladder response to different types of stimuli or insult. Current research strongly suggests an underlying inflammatory process, as demonstrated by increased serum levels of C-reactive protein and proinflammatory cytokines, although the precise cause for this is not well understood [9,10]. Prevalence inside a population can be variable; the reported prevalence is 52–500/100,000 in the female and 8–41/100,000 in the male population [11]. The key to the prognosis of IC is a careful history with identification of the characteristic symptoms, especially severity of pain and soreness [3]. IC normally affects the patient during the storage phase and appears to originate within the bladder; it is described as an imperative urge for bladder filling with increasing suprapubic ache that extends all through the pelvis, relieved by voiding. Different descriptions of the sensation include ‘pressure’, ‘burning’, ‘sharp’, and ‘discomfort’ [2,5,7]. Due to multifactorial etiology, numerous hypotheses have been proposed, such as urinary tract infection, surgery, or viral illness, although the most recognized is a progressive weakening of the bladder wall lining, consisting of glycosaminoglycans such as hyaluronic acid, chondroitin sulphate, heparan sulphate, and dermatan sulphate [12]. Biopsies analysis shows increased urothelial permeability due to diminished GAG levels and ultrastructural abnormalities characterized by a loss of tight junctions and adhesive junction proteins [10]. If a physiological situation occurs, GAGs cover the urothelium and play a crucial role in protecting the bladder and preventing the adhesion of pathogenic bacteria to it [12]. Unlike this, the damaged protective layer lets in the infiltration of harmful urine components with inflammatory action into the underlying layer, resulting in the consequent activation of mast cells [13] and the overexpression of angiogenic factors, such as vascular endothelial growth factor (VEGF) and platelet-derived endothelial cell growth factor (PD-ECGF) in the submucosa [14]. Furthermore, disruption of the GAG layer causes the release of adhesion factors such as transforming growth factor-beta (TGF-β) and CD44 that, during the wound-healing process, bind with angiogenic factors, via heparin-binding, and become solidified. Therefore, it is reasonable that this overexpression of angiogenic factors is involved in tissue fibrosis and the consequent observed bladder atrophy [15]. In addition to TGF-β, also nerve growth factor (NGF) is increased in IC/BPS patients [16]. Bladder pain and urinary frequency are the most important complaints observed in IC/BPS patients, but there are others who have a broader spectrum of problems, including associations of IC to allergy, Hashimoto′s thyroiditis, rheumatoid arthritis, ankylosing spondylitis, irritable bowel syndrome, vulvodynia, fibromyalgia, chronic fatigue syndrome, anxiety disorders, and depression as a comorbid condition [17,18]. It would be useful to acknowledge such associations in the patient’s history in order to better characterize pathology and potential treatment. Such a stress implication in IC/BPS has also been shown at the molecular level, where the involvement of the stress-response corticotropin-releasing hormone receptor (CRHR) was found in the bladder from IC/BPS patients [19]. Indeed, the data collected revealed that CRHR1 expression was mainly located in the submucosa, while CRHR2 expression was mainly in uro-epithelial cells, with significant differences between HIC and NHIC patients [20]. In addition, data in mice showed that acute stress induces bladder vascular permeability and VEGF release that depends on CRHR2, confirming that CRH and VEGF might participate in the pathogenesis of IC/PBS [21]. All these data are consistent with the evidence that CRH, CRH-related peptides, and their receptors are associated with the pathophysiology of many stress-related disease states [22,23], including IC/PBS.

### 2.3. Histopathology and Nitric Oxide Role in IC/BPS

Even though IC patients show a high incidence and degree of denuded epithelium, ulceration, and submucosal inflammation, none of these findings can be considered pathognomonic [24]. Other microscopic findings are urothelial vacuolization and detachment, mucosal infiltrates of lymphocytes, plasma cells, neutrophils, and eosinophil granulocytes, as well as an increase in submucosal mast cell numbers, that are supposed to be the first player in this disease [25,26]. Mast cells can be activated by several agents, leading to the release of inflammatory mediators, with or without degranulation [27]. Moreover, their hyper-activation stimulates unmyelinated C fibers, leading to bladder pain and neuro-peptide release, causing secondary damage to the mucosa and fibrosis of the submucosa [28,29,30]. Although it is still unresolved how these differential mast cell responses are controlled, it is widely recognized that mast cell are a major target of immune CRH that it is secreted peripherally at inflammatory sites (peripheral or immune CRH), influencing the immune system directly through local modulatory actions [31,32]. It is reasonable to hypothesize that the different components of the cell play various roles, although they are currently poorly characterized [33]. There are studies suggesting that T and B cells are associated with different clinical features of IC; moreover, there are marked differences in lymphocyte populations in classic as opposed to nonulcer disease, while macrophage type M1 have a possible role in the massive production of nitric oxide (NO) seen in Hunner-type disease [34]. NO is a physiological mediator that regulates vascular function and inflammation and acts as a sensory neurotransmitter also in the bladder [35]. But, because of the excessive production of ROS at the inflammation site, NO may be converted into proinflammatory and cytotoxic substances through the formation of reactive nitrogen species (RNS) products [36,37]. It has been proven that patients with nonulcerous IC do not show elevated levels of NO compared to those with classic IC [38]. Moreover, it was reported that the NO concentration reduced in patients treated with steroids and that it correlated with a decrease in symptom score in these patients [39].

## 3. IC/BPS Treatment

### 3.1. Conservative Treatments

Dietary and lifestyle modifications together with specific activities should be the initial treatment for IC/BPS. Indeed, although there are different treatments, to date, no effective conservative treatment specific for IC has been identified yet [4,40]. Pentosan Polysulfate Sodium (PPS) is the only FDA-approved drug specific for IC/BPS treatment, but some evidence shows a progressive, vision-threatening macular condition associated with long-term PPS use [41]. In addition, not all available treatments are effective for all IC patients. Consequently, it is necessary to tailor treatment for each patient according to symptoms. Patients usually receive several treatments (or combinations of treatments) until desirable symptom relief is achieved, and it is often necessary to wait weeks or even months for the satisfactory relief of pain symptoms. Even with successful treatment, the condition may not be cured. Therefore, in order to elucidate efficiency and safety in patients with IC/BPS, current clinical studies based on case reports are not sufficient, and large, multicenter, long-term, randomized clinical trials are warranted [42]. In the next paragraph, we will discuss some investigational regenerative approaches that, based on the molecular features of IC/BPS described above, could work in order to aim at the regeneration of connective tissues and associated epithelia, as well as for pathologies of other organs and systems.

### 3.2. Investigational Therapies

#### 3.2.1. Hyaluronic Acid

The restoring of the protective layer of GAGs is an important aim of IC/BPS treatment. Indeed, among the molecules with proven efficacy in IC/BPS treatment, there are glycosaminoglycans that include hyaluronic acid (HA). HA is a nonsulfated mucopolysaccharide based on two sugars, D-glucuronic acid and N-acetyl-D-glucosamine, bonded with alternating β-1,4 and β-1,3 glycosidic bonds [43]. It exists in the form of many different molecular weights (MWs), with a variable half-life and is characterized by interesting viscoelastic and rheological behavior due to its polymeric and polyelectrolyte properties [12,44,45]. HA is a component of numerous fluids and biological tissues, where it is involved in either structural or biological processes such as pathogen/viral defense, coagulation, inflammation, wound healing, cell proliferation, cell adhesion, cell migration, morphogenesis, tissue integrity, tissue flexibility, and lubrication [12,44,45,46]. HA, as described before, is an important component of the GAG layer that increases in the urine of patients with BPS, thus demonstrating a disruption of the GAG barrier at the bladder level [47]. When this barrier decreases or becomes absent, it allows for the diffusion of urinary potassium ions into the bladder wall, resulting in the stimulation of C-type nerve fibers, as already described. Moreover, HA enhances the secretion of enzymes, leading to increased GAGs production, and it is able to alter epithelial permeability stimulating of the expression of tight junction proteins [12]. The immunomodulatory effect of HA is well known and described [48]: HA fragments use both Toll-like receptor (TLR) 4 and TLR2 as well as CD44 to stimulate inflammatory genes in inflammatory cells. HA and the related HA-binding proteins regulate inflammation, tissue injury, and repair through regulating inflammatory cell recruitment, the release of inflammatory cytokines, and stem cells. HA exerts an anti-inflammatory effect by decreasing immune cell infiltration into the urothelium and inhibits bladder mast cell degranulation [49,50]. In addition, high-molecular-weight HA (HMW-HA) may stimulate the production of immunosuppressive M2 macrophages [51].

Over three decades, the intravesical instillation of HA alone, or in combination with chondroitin sulfate, has demonstrated its usefulness in the treatment of IC/BPS, as tested by several clinical trials in terms of symptoms and pain relief [10,52,53,54,55,56,57,58], and long-term symptom remission for up to 5 years of follow-up [59,60] (Table 1). However, there are limited reported data regarding the glomerulation changes before and after HA instillations. A retrospective study in a small group of patients with IC showed that HA repaired bladder glomerulation, although no strong correlation was found between the initial glomerulation grade or changes in glomerulation grades with clinical symptoms [61]. On the other hand, it is known that during injury, HMW-HA is catabolized in lower-molecular-weight (LMW-HA) HA from endogenous and/or microbial hyaluronidases (in the case of infection), mechanical forces, or even oxidation [62]. LMW-HA would have the ability to act as damage-associated molecular patterns (DAMPs), promoting the activation and maturation of immune cells, the release of proinflammatory cytokines such as interleukin 1 beta (IL-1β), and tumor necrosis factor α (TNF-α), increasing the expression of chemokines and cell proliferation [62], as observed also in the fibrotic lung [63]. Therefore, beyond the reparative role of the GAG layer, it would be worth investigating the immunomodulatory function of HA, and the function played by its different molecular weights in cellular proliferation and differentiation, as already showed in other organs [64,65].

#### 3.2.2. Derived Mesenchymal Stem Cells

Understanding the molecular mechanisms underlying stem cells’ (SCs) action in bladder disease is important to improve their therapeutic effect against IC/BPS. Although it was initially thought that SCs transplantation into the bladder might fill in the damaged epithelium and neurons [71], more recently, the hypothesis according to which transplanted SCs might provide therapeutic benefits through the paracrine release of anti-inflammatory, proangiogenic, antiapoptotic, and/or antioxidative factors, including exosomes, was accepted [72,73,74]. Furthermore, it has become clear that these paracrine bioactive factors lead to the recruitment of endogenous SCs to damaged tissues [75]. Then, mesenchymal stem cells (MSCs) also have immunomodulatory properties; therefore, they can be transplanted allogenically or xenogenically into immunocompetent recipients without the use of immunosuppressants [76]. As we already reviewed [77], in 2017, Kim et al. discussed the possibility of using hESC-derived multipotent mesenchymal SCs (M-MSC) in IC/BPS in animals [78]. In their study, they showed the ability of M-MSCs therapy in ameliorating defects in bladder voiding function and reducing visceral hypersensitivity and the superior therapeutic potency of M-MSCs compared to adult bone-marrow-derived MSCs [78]. The same evidence was also confirmed by two other works based on a rats model of cystitis and IC/BPS [79,80]. However, although preclinical studies point to SCs as being favorable in IC/BPS treatment, the therapeutic mechanism of SCs is yet to be completely understood, and results deriving from animal studies should be carefully interpreted and critically evaluated before designing clinical trials [42,81]. Furthermore, the paracrine effects of transplanted SCs seem to be more important because of their stimulation of the host’s own SCs and adjacent cells [71,82]. In 2022, Shin et al. reported the 12-month follow-up of three IC/BPS patients treated with MSCs derived from hESCs. VAS and their lesions were greatly improved, and one lesion in the first patient became unidentifiable under cystoscopy [66] (Table 1). As we already reviewed [77] in another bigger clinical trial, Lander et al. [67] (Table 1) investigated the effect of the combined intravenous and local injection of autologous stromal vascular fraction (SVF) into the pelvic floor in 91 women and 18 men with IC/BPS. Most patients in this study showed limited clinical responses after receiving multiple various medications and procedural interventions. Adipose-derived stromal cells (ASCs) that persist within the SVF of fat depots during adulthood represent the adipose-resident pool of MSCs [83], the potency of which may extend beyond mesenchymal phenotypes as they are able to differentiate into derivatives of all three germ layers [84,85]. ASCs were also used in refractory hemorrhagic cystitis showing clinical efficacy [86]. SVF, which is a source of mesenchymal cells, endothelial precursor cells, T regulatory cells, macrophages, smooth muscle cells, pericytes, and preadipocytes, may reduce the level of inflammation, as indicated by the lower expression of inflammatory cytokines and higher expression of anti-inflammatory cytokines (lower IL-6 and TNF-α expression, and higher IL-10 expression and M2 macrophage numbers) [87,88,89]. SVF, when interacting with lymphocytes, displays potent immunosuppressive and anti-inflammatory effects, negatively regulating T cell, B cell, NK cell proliferation, and maturation of dendritic cells [90]. Moreover, as reported by Kim et al., SVF contains insulin-like growth factor (IGF), pigment epithelium-derived factor (PEDF), secreted super-oxide dismutase (SOD), and glutathione peroxidase (GPX) as protective agents against free radical [91]. MSCs, that are part of the VSF as described, significantly reduce urine storage pressure, hypertrophy, and fibrosis in the bladder via their immunomodulatory effects after 4 weeks, as showed in an animal model, with a mechanism that reduces the TNF-α levels and increases the IL-10 and VEGF levels [92]. In the study carried by Lander et al. [67], 71.5% of patients reported that they felt better after SVF treatment. Patients also reported significant decreases in overall mean pain scores, significant decreases in mean Urgency/Frequency Patient Symptom Scale (PUF), and improved O′Leary–Sant symptom (OSS) and bother scores at 1 year. This evidence proves that SVF could be a good and safe autologous personalized regenerative strategy to alleviate IC/BPS. However, the study also showed some limitations, such as the lack of a control group, inconsistent transplantation cell numbers, inconsistent cell injection routes in male patients, lack of information on the molecular mechanisms of SVF therapy, and the absence of long-term data [67].

#### 3.2.3. Platelet-Rich Plasma

Platelet-rich plasma (PRP) carries bioactive molecules such as growth factors and cytokines [93] that can stimulate the healing of soft tissue and joints. For its properties, PRP would offer an effective treatment modality to improve the healing of bladder mucosa injuries and to reduce neuropathic pain [94,95,96]. However, the precise mechanism of bladder healing in IC/BPS mediated by PRP is not clear yet. As discussed above, neurogenic inflammation cascade, observed in IC, would be a consequence of urothelial barrier dysfunction [95]. As a result of this barrier leaking, chronic inflammation and bladder fibrosis would lead to bladder pain and a small bladder capacity [79]. Therefore, as also observed in the wound-healing process, we can speculate that PRP injected into the bladder might release several molecules that foster the proliferation of cells, their migration to the damaged urothelium, subsequent cell differentiation, and angiogenesis [97,98]. PRP may also activate additional inflammatory signaling by its content of cytokines and leukocytes, switching to an anti-inflammatory phenotype and releasing anti-inflammatory factors and an anxiolytic factor as serotonin that eliminates refractory neuropathic pain [81,99]. As already mentioned in our previous review [77], in a prospective clinical trial (ClinicalTrial.gov: NCT03104361; IRB: TCGH 105-48-A.), Jhang et al. [68,69,100] (Table 1) investigated the changes in urinary markers after PRP treatment. In their study [101], 40 patients (37 women and 3 men) with IC/BPS, who had previously failed to conventional treatments, received four injections of PRP at monthly intervals. In detail, the authors evaluated the urine levels of thirteen functional proteins, growth factors, and cytokines at baseline and after the fourth PRP injection. They also analyzed the clinical parameters global response assessment (GRA) score as the primary endpoint at 3 months after the last injection of PRP and OSS score, visual analog scale (VAS) score, micturition frequency, nocturia, functional bladder capacity, posturination residual (PVR), and maximum flow rate as the secondary endpoints at baseline and at 3 months after the last injection. They observed a significant decrease in GRA and symptom score post-treatment, as well as a significant decrease in OSS, VAS, urinary frequency, and nocturia. Urinary levels of NGF, MMP-13, and VEGF also significantly decreased post-treatment, whereas PDGF showed an increase at the fourth PRP treatment, compared with baseline, together with the bladder capacity [101]. The same authors, in another recent clinical trial, showed a significant improvement in urothelial tight junction defects in patients with refractory nonulcer IC/BPS [70] (Table 1). Therefore, considering that proteoglycan deficiency with the consequent increase in urothelial permeability is one of the most relevant pathophysiological mechanisms of IC, the use of PRP has a consistent rationale and may be clinically beneficial as well as relatively cheap and easy to prepare. As the understanding of the molecular mechanism underlying PRP action on the bladder is hindered by poor histological and ultrastructural studies, some data could be obtained from what has emerged on elderly human skin, which has some structural analogies with interstitial cystitis, such as those linked to an increase in the fibrous component of the connective tissue [102,103]. Moreover, the modality of the preparation of PRP assumes a fundamental role in the pathophysiologic environment of IC. For example, the use of acid citrate dextrose-A (ACD-a) can induce a severe inflammatory reaction. D-Dextrose interacts with the cytokine TNF-α, with an increase in the inflammatory cascade.

#### 3.2.4. Comparative Analysis among HA, SCs, and PRP

The major advantage of the use of PRP for therapeutic application in IC/BPS is the immediate preparation of PRP, which does not require any GMP facility as in the case of SCs manufacturing. Because PRP does not require any substantial modification, it is considered safe and natural in opposition to SCs expansion. Autologous PRP and SCs do not elicit immune response since the preparations are from the same person. In general, the use of PRP does not have major demerits; however, under certain circumstances, PRP applications can result in injection site morbidity, infection or injury to nerves or blood vessels, as well as scar tissue formation and calcification at the injection, and ache or soreness at the site of injection [104]. Moreover, it is important to note that the use of PRP is discouraged in individuals with a history of heavy smoking, drug, and alcohol use, in patients diagnosed with platelet dysfunction syndromes, thrombocytopenia, hyperfibrinogenemia, hemodynamic instability, sepsis, acute and chronic infections, chronic liver disease, anticoagulation therapy, chronic skin diseases, or cancer, and those with metabolic and systemic disorders. This is in order to avoid the complications associated with PRP-based treatment [104].

SCs, as isolated MSCs, have a much more potent regulatory role in the immune system. Compared to a PRP-based approach, SC therapy is very promising in the treatment of many degenerative diseases where PRP is not suitable [104]. Stem cells can differentiate into new bladder cells, replacing damaged tissue and restoring lost functions. They can also modulate the immune response, as previously described. Although SC has advantages over the PRP-based approach in regenerating damaged tissue, there are certain concerns in using SC. In fact, SCs capabilities of self-renewal and differentiation are highly influenced by their local environment, making it difficult to interpret how an expanded population of MSC may behave in vivo. The isolation, culture, and characterization of SCs take several weeks and have to be carried out in GMP facilities by highly qualified professionals. Therefore, to avoid some complications of employing SCs, it is possible to use surgically procured stromal vascular fraction (SVF) derived at the point of care. SVF is an autologous biologic product derived from lipo-aspirate that can replace purified SCs. SVF as PRP is easy to obtain and prepare, without the need for any substantial manipulation. As already described, the fraction contains MSCs together with other cells that may reduce the level of inflammation and protect against free radicals. To date, clinical trials with a longer follow-up are missing; therefore, there are still few data regarding the potential long-term risks associated with SVF therapy. In general, long-term studies and observations will be necessary to investigate the long-term effects of cells and PRP therapies, including the negative effects [105].

HA has been used for a longer amount of time compared to SCs and PRP in the treatment of IC/BPS, with good result being obtained in terms of long-term symptom remission up to 5 years follow-up. However, its employment could be indicated more towards improving bladder pain rather than restoring physiological functions [58]. Thus, a combination of HA with either PRP- or MSCs-based therapy would improve healing capacity through the regenerative and antioxidant capacity of the factors released by platelet and cells.

## 4. Discussion

**Figure 1:**
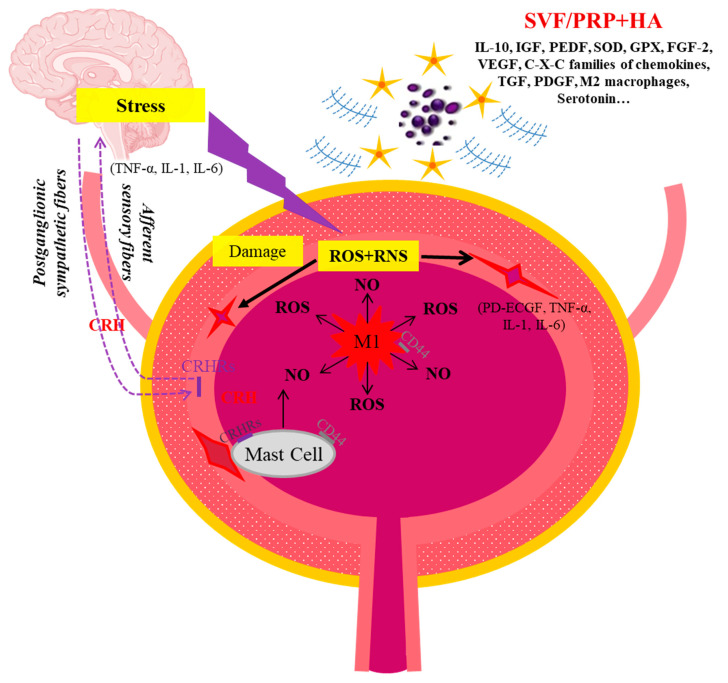
Synergetic effect of SVF/PRP + HA on the anti-inflammatory action, immune-modulation, and regeneration of bladder mucosa and epithelium. CRH: corticotropin releasing hormone. CRHR: corticotropin releasing hormone receptor. M1: macrophages M1. NO: nitrogen oxide. RNS: reactive nitrogen species. ROS: reactive oxygen species.

Through the studies considered, it seems obvious that oxidative stress is an underlying mechanism in the pathophysiology of IC/BPS, where inflammation and biological damage may be produced as a consequence of an overwhelmed antioxidant capacity, as also observed in aged and fibrotic tissue [106,107], although it is still unknown whether high levels of NO have a protective or damaging role in this disease. The presence of CRHRs in the mucosa and uro-epithelial cells of the bladder and their dysregulation in patients with IC/BPS suggests that CRH signaling may be associated with IC/BPS symptoms, with a consequent important role played by stress as well in this pathology. In fact, during a chronic harmful challenge, in response to inflammation, the activated HPA axis modulates, through CRH release, the immune response via glucocorticoid activity, with an associated strong imbalance in redox homeostasis [108]. As described before, mast cells that are increased in IC/BPS patients are the main target of immune CRH, and mast cells may activate other immune cells, such as macrophages. Accordingly, this imbalance and the consequent increase in oxidant production may induce the conversion of NO, which is produced by mast cells and macrophages as a toxic defense molecule against infectious organisms, into proinflammatory and cytotoxic substances, through the formation of RNS products. This overproduction of ROS and RNS produced by immune cells would lead to cell membrane destruction with consequent uro-epithelial damage (Figure 1). Data deriving from the few studies carried out on humans, described in this review, show that stem cells and PRP may repair the damage observed in IC/BPS. There are several suggested mechanisms used by stem cells to repair. One common mechanism is by cell formation following direct stem cell engraftment and trans-differentiation guided by cellular damage milieu [67]. Another mechanism is through the ‘paracrine’ effect, wherein stem cells use complex cellular signaling systems, which may lead to increased angiogenesis, the promotion of cell survival, and the prevention of cellular apoptosis in damaged tissues [67]. Growth factors released by stem cells or PRP communicating between healthy stem cells and damaged target cells may modulate the microenvironments of the damaged target tissues, making them more favorable for the regeneration process and enhancing the regenerative potency of the damaged tissues [75]. Cell signals may also be mediated by exosomes deriving from engrafted stem cell or PRP [109]. Exosomes contain proteins, mRNA, miRNA, and other genetic material. Moreover, recent evidence has shown that a paracrine effect can also be played by the direct transfer of mitochondria from healthy stem cells to damaged cells to restore oxidative phosphorylation and rejuvenate the energy production capabilities of unhealthy and damaged cells [110]. Therapy based on SVF may reduce the level of inflammation modulating the expression and suppression of various cytokines, the conversion of the macrophage phenotype, and the regulation of T cells [111]. Moreover, SVF contains IGF, PEDF, SOD, and GPX as protective agents against free radicals [91]. All these activities described may assist in reducing ROS-induced damage. However, adipose stem cells could be inflamed or aged. As for PRPs, they play an important role in tissue repair, mainly due to many growth factors, such as PDGF and IGF-I. PRPs also contain pain-reducing factors such as serotonin. On the other hand, HA plays an important role both in the protection and restoration of urothelium homeostasis and an immune-modulatory role through binding to CD44 expressed by mast cells and macrophages [112]. Recent evidence has also highlighted the role of LMW-HA in the process of cell proliferation and differentiation. Therefore, in accordance with the IC/BPS features described, we suggest that the combination of both adipose tissue-derived MSC and HA, or PRP and HA, could have the ability to accelerate epithelialization, to induce angiogenesis, to stimulate fibroblasts, and to inhibit a local and systemic stress oxidative response, as well as to modulate the immune response. A recent publication showed that the combination of HA and MSCs had a great therapeutic effect for nerve regeneration engineering through the ability of HA not only to maintain the greatest stemness properties and regulate the neurogenic differentiation ability of MSCs but also to induce the least immune response [113]. As an example of this synergy between MSCs and HA, it has been shown that IL-10, that is expressed by MSCs, in turn stimulates fibroblasts to secrete HMW-HA to prevent collagen deposition and inflammatory macrophage polarization [114]. In another recent work, a combination of adipose tissue-derived MSCs and autocross-linked HA gel was used for the treatment of intrauterine adhesion in a rat model [115]. Moreover, MSCs derived from bone marrow (BMSCs) combined with HA showed good results in terms of articular cartilage repair in a canine model [116], while the combination of PRP with HA in the urology field has not shown a synergistic effect on an IC/BPS rat model [117], as observed in the orthobiology field [118]. Moreover, ASCs are promising because they are easy to collect and, as autologous, can be used in one-step procedures. Furthermore, they can be dissociated mechanically avoiding enzymatic treatments that are not allowed by many Regulatory Medicine Agencies [119].

Although there are still few and only small-sized clinical trials based on regenerative approaches, the evidence produced so far would indicate that the combination of SVF or PRP with HA could potentially be an effective alternative treatment for IC/BPS and other diseases of the integumentary system, or a better treatment for a subgroup of patients. A last consideration is that since IC/BPS is a complex and heterogeneous disease, many patients fail treatment because the therapies used target specific molecular mechanisms. Instead, cell therapy, PRP, and their combination with HA could be more effective as they aim to harness the body’s own healing ability to restore lost function and to re-establish normal function.

## 5. Conclusions

The present review shows that, although limited, the treatment of interstitial cystitis with regenerative approaches appears thrilling and promising for future developments related to the improvement in treatment protocols. Unfortunately, regenerative medicine in IC/BPS still lacks standardized procedures and clear international guidelines that limit the variability of regenerative protocols, which generally appear to be more complex than pharmacological treatments, and which sometimes require multidisciplinary team interventions. Nevertheless, despite these limitations, it is evident from the results obtained so far from the few clinical trials conducted that both stem cells and PRP treatments have the potential of wider clinical applications in the near future, in combination with hyaluronic acid, which has been used for years. In fact, considering the multifactorial nature of IC/BPS, we think that the combination of regenerative therapies with existing treatments would bring about potential benefits in the treatment of IC/BPS in a larger number of patients. The data presented in the literature, albeit limited, also demonstrate how interstitial cystitis represents a valid model for the molecular study of innovative therapies aimed at the regeneration of connective tissues and associated epithelia. Therefore, the results obtained to date can be useful for the development of regenerative procedures, as well as for pathologies of other organs and systems. However, due to the complexity of the regenerative approaches of structures involving connective tissue and epithelia, more complete knowledge of the aspects related to both stem cells and PRP treatment is required. This knowledge may be reached from new experimental evaluations, obtained through molecular and cellular biology techniques, in parallel with more clinical trials with larger sample sizes that will demonstrate robustly the safety and efficacy of these innovative approaches. Only an increase in and enlargement of clinical studies will allow for the standardization of outcome assessments, which would facilitate a better comparison and synthesis of the results.

## Figures and Tables

**Table 1:** Summary of investigational therapies based on hyaluronic acid, mesenchymal stem cells, and platelet-rich plasma, found in the text.

Investigational Therapies	Authors	Year
Hyaluronic Acid	Jhang J. et al. [10]	2022
	Morales A. et al. [52]	1997
	Kallestrup E.B. et al. [53]	2005
	Riedl C.R. et al. [54]	2008
	Shao Y. et al. [55]	2010
	Porru D. et al. [56]	2012
	Cervigni M. et al. [57]	2017
	Hung M.J. et al. [59]	2019
	Engelhardt P.F. et al. [59]	2011
	Scarneciu I. et al. [60]	2019
Mesenchymal Stem Cells	Shin J.H. et al. [66]	2022
	Lander E.B. et al. [67]	2019
Platelet Rich Plasma	Jhang J.F. et al. [68]	2019
	Jhang Y.H. et al. [69]	2020
	Lee Y.K. et al. [70]	2022
